# Supplementary material for: Hydrogen trapping and embrittlement in high-strength Al alloys
Source: Nature. 2022 Feb 16;602(7897):437–41. doi: 10.1038/s41586-021-04343-z (PMC8850197; doi:10.1038/s41586-021-04343-z)
Supplement: Supplementary file 1 — This file contains Supplementary Figures 1–4 and Supplementary Table 1. [file 41586_2021_4343_MOESM1_ESM.pdf]

nature portfolio

https://doi.org/10.1038/s41586-021-04343-z

# Supplementary information

# Hydrogen trapping and embrittlement in high-strength Al alloys

In the format provided by the
authors and unedited

## Supplementary Information

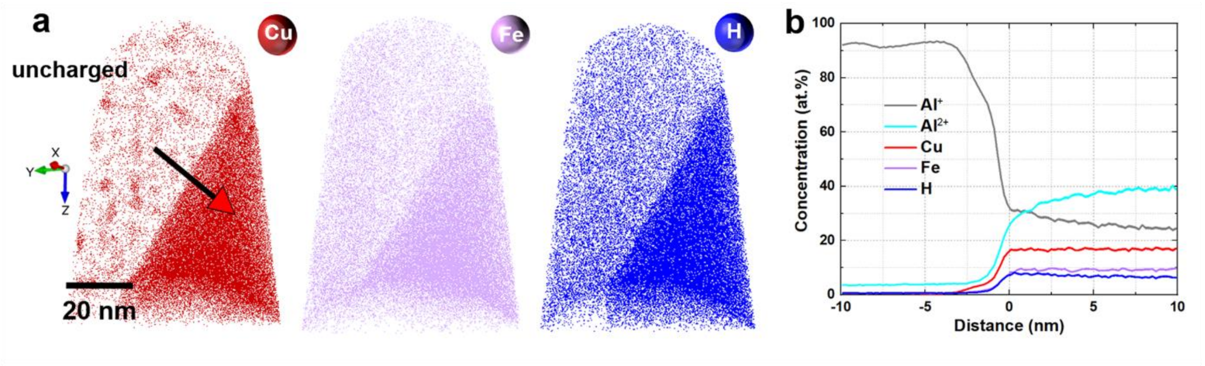


*Fig. 1. APT analysis of a peak-aged Al sample containing $Al_7Cu_2Fe$ intermetallic phase. (a) Atom maps of Cu, Fe, H are presented, with the intermetallic phase visualized by the Cu-Fe enriched region. (b) Composition profile across the intermetallic phase showing H concentration of 9 at.%.*

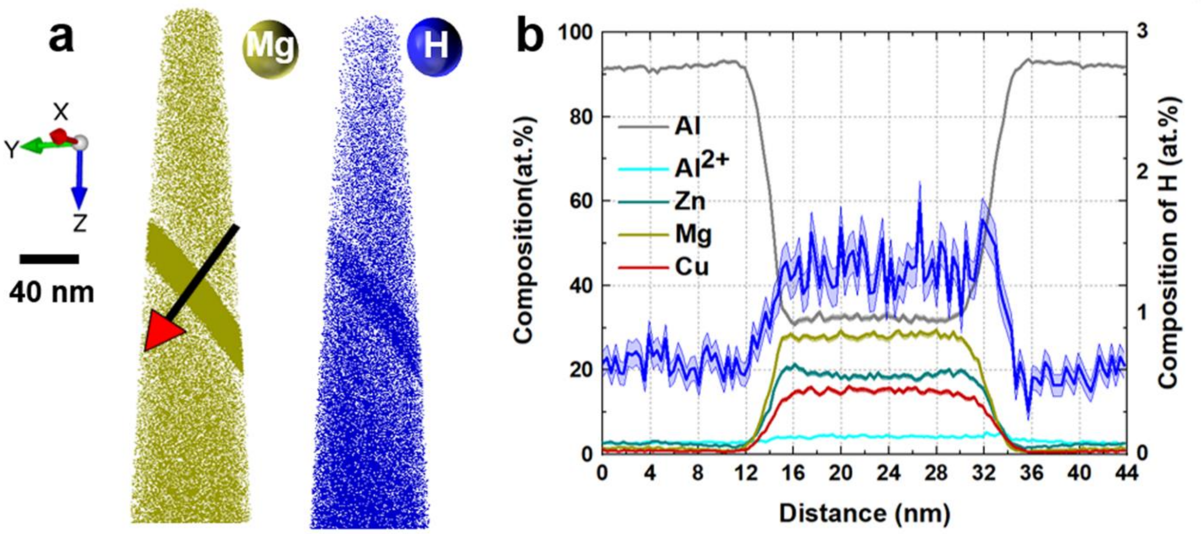


*Fig. 2. APT analysis of a peak-aged Al sample containing T-phase. (a) Atom maps of Mg, and H are presented. (b) Composition profile across the T-phase showing H concentration of 1.5 at.%.*

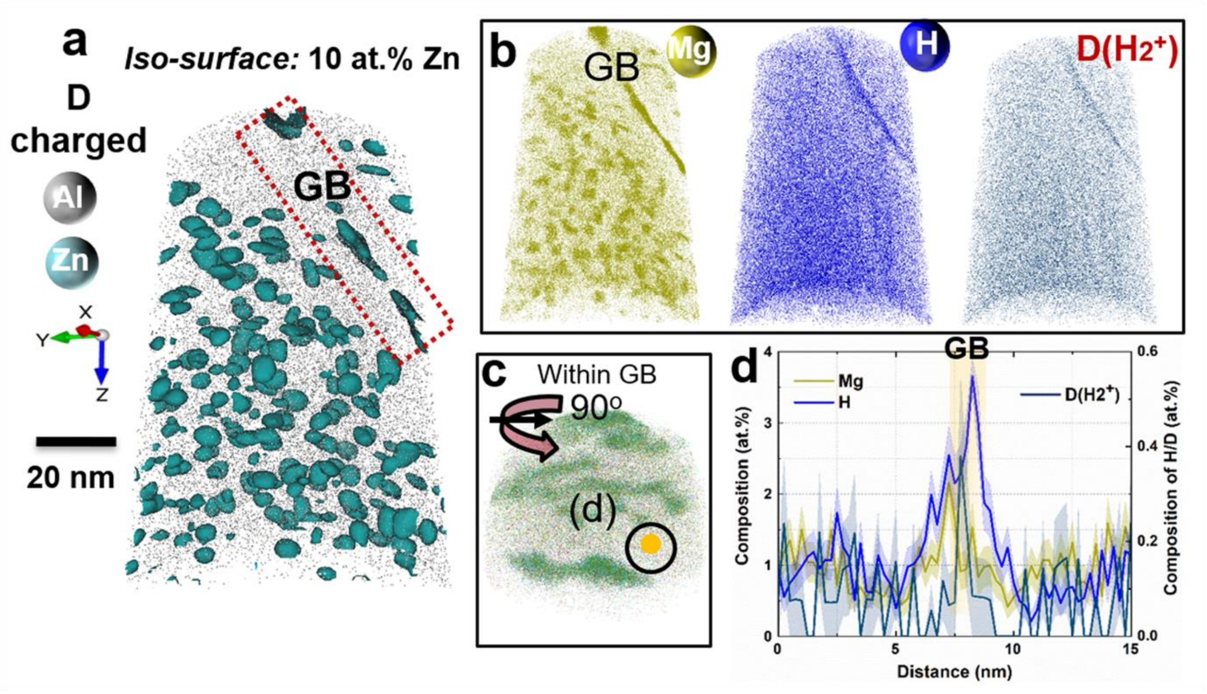


*Fig. 3. APT analysis of a GB in the peak-aged Al-Zn-Mg-Cu sample subjected to D-charging (120 $^{o}$C/24h). (a) A set of isosurfaces highlight the dispersion of fine Zn-rich precipitates in the matrix and of the larger ones at the GB. (b) Atom maps of Mg, H and D($H_2^+$). (c) Solute distribution at the GB plane. (d) Representative solute composition profile across the GB in between of precipitates shown in the region highlighted by the circle.*

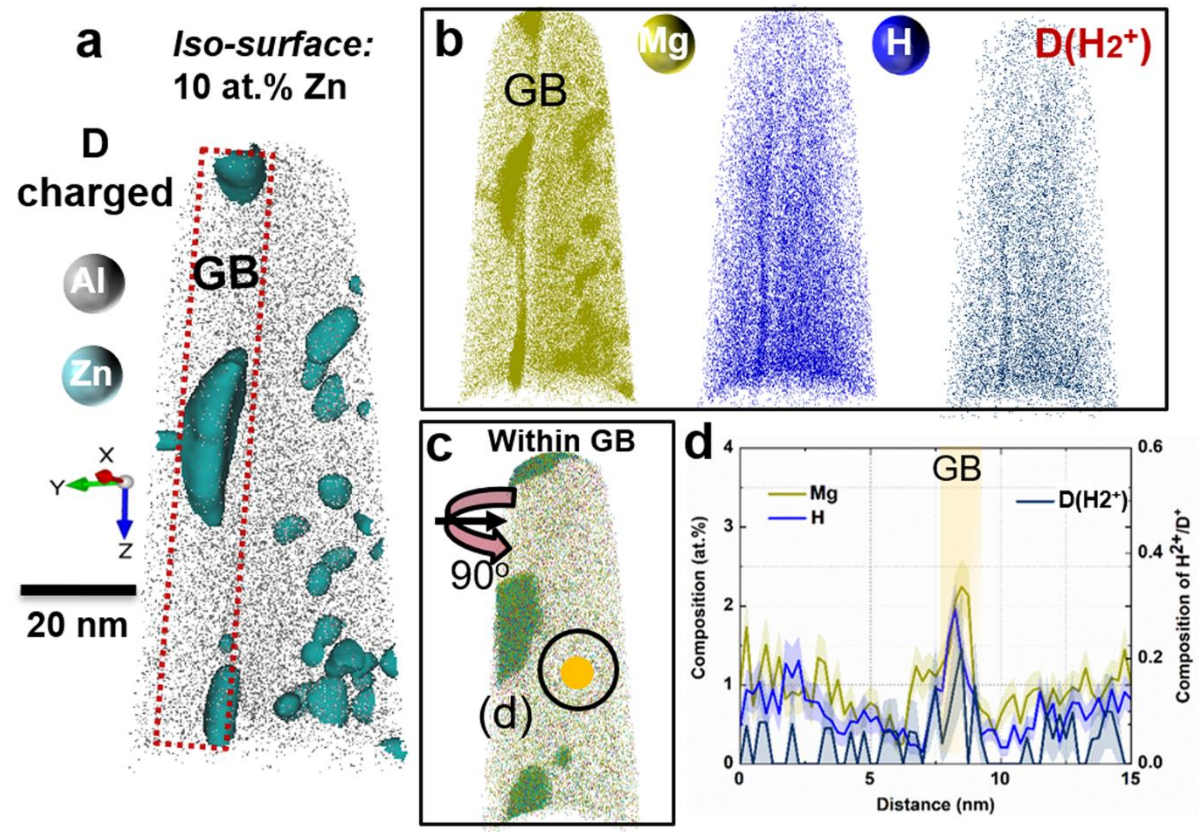


*Fig. 4. APT analysis of a GB in the peak-aged Al-Zn-Mg-Cu sample subjected to D-charging (120 °C/24h). (a) A set of isosurfaces highlight the dispersion of fine Zn-rich precipitates in the matrix and of the larger ones at the GB. (b) Atom maps of Mg, H and D($H_2^+$). (c) Solute distribution at the GB plane. (d) Representative solute composition profile across the GB in between of precipitates shown in the region highlighted by the circle.*

*Table 1*

*H and D composition within the second-phases and grain boundaries in the D-charged and uncharged states.*

| Interface | Condition | H (at.%) | **D (at.%)** | **$C_H/C_D$** |
|---|---|---|---|---|
| Zr-dispersoid | uncharged | 8.50 | 0 | 0 |
| | D-charged | 9.50 | **2.80** | **3.39** |
| S phase | uncharged | 6.82 | 0.03 | 227 |
| | D-charged | 4.20 | **0.12** | **35** |
| Grain boundary | uncharged | 1.37 | 0.10 | 12 |
| | D-charged | 2.42 | **0.31** | **7.81** |
